# Heterologous Antilymphocyte Serum Hastens the Growth of 7,12-Dimethylbenz(α)Anthracene Induced Tumours in Mice

**DOI:** 10.1038/bjc.1973.140

**Published:** 1973-09

**Authors:** C. D. Baroni, R. Scelsi, M. L. Peronace, S. Uccini

## Abstract

The present paper describes the effects of repeated administration of rabbit anti-mouse lymphocyte serum (ALS) or normal rabbit serum (NRS) on tumours induced in Charles-River mice by 7,12-dimethylbenz(α)anthracene (DMBA) given at birth. ALS or NRS were given either at the time of DMBA administration and subsequently at weekly intervals for the first 10 weeks of life, or at daily intervals for 7 days during the first, second, third or fourth week of life. The incidence and histology of the tumours were studied. It was found that treatment of very young mice with ALS greatly reduced the mean survival time of mice and significantly increased the incidence of malignant lymphoma. The incidence of lung tumours was found to be significantly increased in the animals injected with ALS during the second week of life.

Treatment with ALS in the other experimental groups gave results essentially similar to those observed in DMBA control and NRS treated mice.


					
Br. J. Cancer (1973) 28, 221

HETEROLOGOUS ANTILYMPHOCYTE SERUM HASTENS THE
GROWTH OF 7,12-DIMETHYLBENZ(a)ANTHRACENE INDUCED

TUMOURS IN MICE

C. D. BARONI, R. SCELSI, M. L. PERONACE AND S. UJCCINI

From the Institute of Pathological Anatomy II of the University of Rome, 324 Viale Regina Elena,

00161 Rome, Italy, and the Institute of Pathological Anatomy of the University of Pavia, Italy

Received 1 November 1972. Accepted 14 May 1973

Summary.-The present paper describes the effects of repeated administration of
rabbit anti-mouse lymphocyte serum (ALS) or normal rabbit serum (NRS) on
tumours induced in Charles-River mice by 7, 12 -dimethylbenz(a)anthracene (DMBA)
given at birth. ALS or NRS were given either at the time of DMBA administration
and subsequently at weekly intervals for the first 10 weeks of life, or at daily intervals
for 7 days during the first, second, third or fourth week of life. The incidence and
histology of the tumours were studied. It was found that treatment of very young
mice with ALS greatly reduced the mean survival time of mice and significantly
increased the incidence of malignant lymphoma. The incidence of lung tumours
was found to be significantly increased in the animals injected with ALS during
the second week of life.

Treatment with ALS in the other experimental groups gave results essentially
similar to those observed in DMBA control and NRS treated mice.

ANTILYMPHOCYTE serum (ALS) and
other immunosuppressive agents have
been described as factors enhancing tumour
incidence, this description being based on
evidence of medical therapy of human
patients as well (Allison, 1970). There
are reports of the effects of ALS on
animals treated with chemical (Fisher,
Soliman and Fisher, 1969; Balner and
Dersjant, 1969; Woods, 1969; Rabbat and
Jeejebhoy, 1970; Wagner and Haughton,
1971; Haran-Ghera and Lurie, 1971) or
viral carcinogens (Allison, Berman and
Levey, 1967; Hirsch and Murphy, 1968;
Law, Ting and Allison, 1968; Vandeputte,
1969; Burstein and Allison, 1970).

While there is evidence for an in-
creased susceptibility to the action of
oncogenic viruses following ALS treat-
ment, it was not indisputably evident that
ALS had the same enhancing effect on
chemically induced tumours. In addition,
there is relatively little in the literature on
the effects of ALS on lymphoma and other
tumours induced by 7,12-dimethylbenz-

(oc)anthracene (DMBA) given at birth in
mice. In a previous paper (Baroni et al.,
1972) we described the effects of a single
administration of ALS and normal rabbit
serum (NRS) on tumours induced by
DMBA given at birth in mice. Since both
ALS and NRS seemed equally to influence
the incidence of the tumours, we thought
it worthwhile to study the possible effects
of repeated injections of ALS and NRS
on tumours induced by DMBA given at
birth.

MATERIAL AND METHODS

Charles-River mice from the colony of this
Institute were used in the present experi-
ments. They are derived from a stock
obtained in 1968 and since then maintained
in our laboratory by brother x sister mating.
Newborn mice were injected subcutaneously
with  7,12-dimethylbenz( a)anthracene
(DMBA) (Eastman Organic Chemicals) ac-
cording to a procedure already described
(Rappaport and Baroni, 1962).

C. D. BARONI, R. SCELSI, M. L. PERONACE AND S. UCCINI

The preparation, titration and character-
istics of ALS and NRS used in the present
study have been described previously (Baroni,
Kimball and Wagar, 1969; Baroni et al., 1972).

The mice were divided in 5 main groups
as follows: Group 1 treated with DMBA at
birth; Group 2 treated with DMBA at birth
and with 10 intraperitoneal injections of
0-10 ml of ALS at weekly intervals (first
injection at birth); Group 3 treated with
DMBA at birth and with 7 intraperitoneal
injections of ALS at daily intervals according
to one of the following schedules: 0 05 ml of
ALS from birth to Day 7 (Group 3A); 041 ml
of ALS from Day 8 to Day 15 (Group 3B),
0.1 ml of ALS from Day 16 to Day 21
(Group 3C), or 01 ml of ALS from Day 22
to Day 29 (Group 3D); Group 4 treated with
DMBA at birth and with NRS given as
designed for ALS in Group B to parallel
hosts; Group 5 treated with DMBA at birth
and with NRS given as designed for ALS in
Groups 3 to parallel hosts (Groups 5A, 5B,
5C, 5D).

All mice were checked weekly and ob-
served until death occurred. Few of them
were killed when moribound. The incidence
and histology of the tumours have been
determined.

After allowance for the effect of mortality,
the chi-squared distribution on one degree of
freedom has been calculated in order to
assess the possible difference in tumour
incidence among the various experimental
groups.  Since spontaneous incidence of
tumours is remarkably low in our colony of
Charles-River mice, these data are not
reported.

RESULTS

Table I shows tumour crops and sur-
vival rates. Treatment of very young mice
withALS (Group 3A) or chronic administra-
tion of the serum for 10 weeks (Group 2)
apparently reduced the mean survival time.
On the other hand, administration of
ALS in the other experimental groups
had less effect on mortality. Table II pre-
sents the expected malignant lymphoma,
lung and subcutaneous tumour crops assu-
ming that DMBA, ALS and NRS treat-
ments are equally carcinogenic. In Table
III are shown the observed and expected
tumours for all periods considered in Tables

I and II. In Table IV the various experi-
mental groups are compared with each
other after allowance for the effect of morta-
lity. It is now clear that treatment with ALS
increases the number of malignant lym-
phomata only if the antiserum is adminis-
tered during the first week of life (Group
3A). In fact, the comparison of Group 1
with Group 3A and of Group 3A with
Group 5A indicates that the increased
number of malignant lymphomata ob-
served in Group 3A is statistically signifi-
cant and due to the ALS treatment.
A statistically significant increased lung
tumour count was noticed in the group
injected with ALS during the second week
of life (Group 3B).

Administration of ALS in the other
experimental groups did not influence the
incidence of tumours induced by DMBA.

DISCUSSION

We have reported in a previous paper
that a single injection of ALS was clearly
incapable either of preventing or increasing
the appearance of tumours induced in
mice by DMBA given at birth (Baroni
et al., 1972). We anticipated that these
negative results could depend on timing,
dosage and number of injections of ALS.
In the present set of experiments we
observed a markedly increased number of
lymphoma bearing mice in the group
given the antiserum for the first 7 days
of life. The fact that such increase of
lymphoma induction was obtained only
in the group given ALS during the first
week of life could be explained, assuming
that malignant cellular transformation in
the lymphoid tissue starts immediately
after DMBA injection (Rappaport and
Baroni, 1962). Thus, ALS given at the
same time and shortly after DMBA
treatment (Group 3A), e.g., during the very
initial phases of malignant transformation
(Rappaport and Baroni, 1962), could be
expected to exert rapid and long-lasting
effects on the immunocompetence of the
host, allowing unimpeded growth of
tumour cells.

222

TUMOURS INDUCED BY DMBA IN MICE

CO: cO> 01 Cb cO m4 _1 CO 0oo

- -00100100 - -- 0

---)0 COO CO O0 e_e

-010 -   10 CO 00 --

CO 0101010101 -   -

CO C - CO 010 -  0 -  - -
10 0- 01 -  CO 0 " -

0110- I> 01 1-0 CO 01 C

cO 10 O < COe CO 0010101s

- - - - 0 CO -001001

C  1  CO o0 0 o   o   t  0  01
o  m o o 0 o0-  o  0

0   CO  CO 0o  oO o_

0 NC- N -  0  O C C 1

01 -   -  CO - -  -  -

0 0 0 0 0 1   C  o0 1 0 1 s o   c

00 00 1 ml o  CO ca o q q

0 1   0 1 -   0 0 0   0   1-

m

*cI

C3)

01

000-0000000

CO CO CO C O 00 o 100010

C   C O  C O  1-   0 0   -   1 0 0 0

-- 1 1 ---  - G _ _  _ _ _

000_0000000
CO CO CO CO 00 X 1000101
00000000000

CL4

0

m

C1)
CL)

CO
c0 W

I =

co

j    Hc

(C)

C3)I

Fe H

I I

-0    H

cc   x

10{

I'

a,  H

CO

17{

(, Bz' E-0

-*  S4

CO 00000000000
; 00000000000

-_00-0--0-00

_

Cc ON o _Nooo o4 -o

-000000000O

00000000000

0 0 0   - _ 0 _ 0 0 0oo 0 0

00 0 0 0 0

o0oooooooo00o

00000000000Q
_ooooooo0001

-0000000000 o C
- 0 - - o0oo100o 01o o

- 0 0   C O  - e   1 0_ - - c _

00000000000

0

w

0

b

0

.4-S

0
00C
n

C2) 4Z

o Z

C)
w

z a

II 1I
O la

FHCO:OO-4OOCOCOOO

O C>   C  MMC

w O 0 000000000 0

C)  I

O   -        -0000000000

C D0000 00C00

m0 0 0 C OX i I 0 1 C   v

0> o  0 0   'c I  CO '  C 0 1 1c

r-  - 0   C O O--q  M

;          ~~~~~~~~11 11 11
?  _   o cqCO c .  N u:   Ca
$:L;;

m
C)
C1)

CO 0=

CO
CO
01
CSi

H

223

it H

C)

li

{ H

0

C)

b-

Co

Co

0m

Co

PA
* C4

0E

Co

?o
0H

C. D. BARONI, R. SCELSI, M. L. PERONACE AND S. UCCINI

E co co 4 c cco e* co eq o U
H 1 00010110000

m  CO.C

0      e   to  t COi  cq  O C1  O

Co EqCDOCiO-CO-C0CO-0

er e- c _o oo _?I c 5 _ t cso_

01    0 0 1 I C NO0-0  I-
cs  oooebocqooooo

~ 0~  k'- CO 101010
E-1      0 0a 10c C o0

CO '1 000000-000
O~~~~~~

Q   C O O 1   CD cO O k -O

F  0 0' O- - - - - O COO

to COO-  k' -k'11C
E 000000000i xo00

01000100 C O CO COO CO

b: ati xxo o n o _= e- U_ o

-00   0 .1  0-00

CD co 0 Ooco C) m  mC

F  00 k- 1000 c - 0100101
X  s00000000000

-10 CO O O -  O O 11   C
H     CO0oQO  0~OO

in 0 k CO tO1  CO c 0 C - N

_ ~ Ct :O O-O10000000D t

C ACA_Ao_ooooo

00 t- 0t to 000 0 00 0 0
O             000

7fO 0 000 O0 000000 C

C OO 101 O  CO CO 10 O

BH o---ooouonoo

00000ul~in00 0000 t

u4 ; 5_ooooocoooo
_    C> ?   C> O O   O O  C) r  cO

F ooo~ooo____

4 -0 CO OOCOCC0 0 01010
_ > XCOC*OCOCiOC,>C;C

o__ooo

0

0

* -4

o5 o o o5 o5 o o o 5 o c
H  0 0 0 00000000C)
EIO O Ooooooo

-s 3 E6 ooooooo

010{ 0000 0 OC 0 0 0

10  0 0 0 C0 0000C

5 o o o o o o o o o o
oooooooooo
o? o o o o, o o o o o o
s .......o o o oo

10 '1 -  CC)  OO.

10 j 0 00 0 0 0 00000

ou:, 4 oooooooooooa

0 ; 8$888888888

0  00 0   C0 10C1011000

oo o o o o o o co o co

E- o o  o  cscse c  o o aq

o B Ev ooo____c') C;os

-~ u

0         0o  0 0 0 o o o 0 o c

o> o o o o o o) o o o o)

F H oo_ooo

fi  0  wco 0 co ooo

o) o o o o oIooC o o
CO~~~~~~~~~~C

Ezooooooo ooocm
0,

W4 Eo o  ooo,oe o; oo e
0  00000000000

_  t

0:W H 00-00  1000

_   o4 t- o o~ O CO CO Co t-- CO0

Hi     10co_C'-CO~4

$:L4                      ~~~~~~1111 11
0        !       ::     !

01COCOCOCO'141010101xo    0           HE~ ~ - -
&4  ~      ~        ~          4 O

224

0

0

C.-
1-

0)

C.)
0)

pq

Co
0)

CO

C.)
H)

?.

m     m
1 E ,

- -- 0 0

TUMOURS INDUCED BY DMBA IN MICE                              225

TABLE III. Observed and Expected Tumours for All Periods (From Tables I and II)

Overall observed         Overall expected             Ratio

Group     MIL     LT      ST       ML      LT      ST       MIL     LT     ST

1        11      13      3       9.04  20-23    2-44     1-21   0(64     1-22
2         15    18      6      11-20   20-66   3-02      1-33   0-87    1-98
3A        9       8      2       2-83    9-38   1-31     3-18    0-85    1-52
3B        8      19      3      10-23    5-37   3-06     0-78    3-53    0-98
3C        7       9      2       5-61    7-82   2-20      1-24   1-15   0 90
3D        6      12)     2       9-63   11-53   2-85     0 62    1-04    0 70
4         6      10      2       7-95   13-18   2-34     0-75    0 75    0-85
5A        7       8      2       8-24   11-34   2-86     0-84    0 70   0-69
5B        9      16      2       9-29   11-90   3-12     09-96   1-34    0-63
SC        6       8      2       7-47   14-12   1-79     080     0 56   111
5D        6      13      2       8-18    8-15   2-75     0 73    1-59    0 72
AIL   Malignant lymphoma.
LT    Lung tumours.

ST    Subcutaneous tumoturs.

TABLE IV. Influence of ALS and NRS on

Tumour Indutction

Chi-squared for

Grouips              Lung    Subcutaneous
compared Lymphoma    tumours    tumours

1 _       ] 713     2 925      3 068
1-4      09- 902    3 350      0 177
2-4       1-767     1 109      2-990
1-3A     13-875     2-786      0*491
1-5A     0-610      3-566      0*386
3A-5A     13-637      1-186      0-621

1-3B    (0910      37 178      0-129
1-SB     0*433      3 - 995    0 529
313-5B     0 495     36-007      0 402

1-3C     0-768      2-761      0-146
1-SC     0-713      5-235      0-152
3C-5C      0 633     2-830      0-042

1-3D     1-792      2-602      0 266
1-5D      1-004     5 469      0-332
3D-SD      1-948      2 905      0 342

1 degree of freedom: 5 % = 3 - 841

10% = 6635

The present results h.ave also demon-
strated that treatment with ALS after
DMBA does not significantly affect lym-
phoma induction. These data suggest that
ALS fails to exert an immunosuppressive
effect upon that part of the immune
system already committed by the newly
formed malignant lymphoma tumour
specific transplantation antigens.

A noteworthy feature was also the
increased incidence of lung tumours ob-
served in the group given ALS during the
second week of life (Group 3B). This
finding could be tentatively explained
assuming that lung cells are less sus-

ceptible to the action of DMBA than
lymphoid cells, and consequently lung
cellular malignant transformation starts
later. If so, the immunodepression follow-
ing treatment with ALS during the second
week of life could account for the increased
number of lung tumours observed in this
group. This in spite of the fact that in
the lung the concentration of the carcino-
gen could be expected to be higher than
in lymphoid tissues receiving their blood
supply via the greater circulation, since
the lung is the first tissue reached by
DMBA injected subcutaneously.

In conclusion, the present results
agree with those of other investigators
(Balner and Dersjant, 1969; Rabbat and
Jeejebhoy, 1970) and indicate that, under
certain experimental conditions, treat-
ment with ALS is capable of enhancing
tumours induced by DMBA given at birtlh.

This work was supported by a grant
to Carlo D. Baroni from the Consiglio
Nazionale delle Ricerche, Roma (C.N.R.)
Research   Contract No.   71.00162.04-.
1153998.  The authors are indebted to
Mrs L. Venturi for technical assistance.

REFERENCES

ALLISON, A. C. (1970) Tumour Development

following Immunosuppression. Proc. R. Soc.
Med., 63, 1077.

ALLISON, A. C., BERMAN, L. D. & LEVEY, R. H.

(1967) Increased Tumour Induction by Adeno-
virtus Type 12 in Thymectomized Mice and

226        C. D. BARONI. R. SCELSI. M. L. PERONACE AND S. UCCINI

Mice Treated with Anti-lymphocyte Serum.
Nature, Lond., 215, 185.

BALNER, H. & DERSJANT, H. (1969) Increased

Oncogenic Effect of Methylcholanthrene after
Treatment with Anti-lymphocyte Serum. Nature,
Lond., 224, 376.

BARONI, C. D., KIMBALL, J. W. & WAGAR, R. D.

(1969) A Histological and Immunological Study
of the Effect of Rabbit Anti-mouse Lymphocyte
Serum (ALS) in Balb/c Mice. Transplantation,
7, 303.

BARONI, C. D., MINGAZZINI, P., PESANDO, P.,

CAVALLERO, A., UccINI, S. & SCELSI, R. (1972)
Immunodepressione e Cancerogenesi Chimica.
I. Effetti di un'unica Dose di Siero di Coniglio
Antilinfociti di topo sui Tumori Indotti nel topo
dal 7,12-dimetilbenz(oc)antracene (DMBA) Som-
ministrato alla Nascita. Tumori, 58, 397.

BURSTEIN, N. A. & ALLISON, A. C. (1970) Effect of

Antilymphocytic Serum on the Appearance of
Reticular Neoplasms in SJL/J Mice. Nature,
Lond., 225, 1139.

FISHER, E. R., SOLIMAN, 0. & FISHER, B. (1969)

Effect of Antilymphocyte Serum on Parameters
of Growth of MCA-induced Tumours. Nature,
Lond., 221, 287.

HARAN-GHERA, N. & LUTRIE, M. (1971) Effect of

Heterologous Antithymocyte Serum on Mouse

Skin Tumorigenesis. J. natn. Cancer Inst.,
46, 106.

HIRSCH, M. S. & MURPHY, F. A. (1968) Effects of

Anti-thymocyte Serum on Rauscher Virus Infec-
tion of Mice. Nature, Lond., 218, 478.

LAW, L. W., TING, R. C. & ALLISON, A. C. (1968)

Effects of Antilymphocyte Serum on Induction of
Tumours and Leukaemia by Murine Sarcoma
Virus. Nature, Lond., 220, 611.

RABBAT, A. G. & JEEJEBHOY, H. F. (1970) Hetero-

logous Antilymphocyte Serum (ALS) Hastens the
Appearance   of   Methylcholanthrene-induced
Tumors in Mice. Transplantation, 9, 164.

RAPPAPORT, H. & BARONI, C. D. (1962) A Study of

the Pathogenesis of Malignant Lymphomas
Induced in the Swiss Mouse by 7,12-dimethylbenz-
(a)anthracene Injected at Birth. Cancer Res.,
22, 1067.

VANDEPUTTE, M. (1969) Antilymphocytic Serum

and Polyoma Oncogenesis in Rats. Transplantn.
Proc., 1, 100.

WAGNER, J. L. & HAUGHTON, G. (1971) Immuno-

suppression by Antilymphocyte Serum and its
Effect on Tumors Induced by 3-Methylchol-
anthrene in Mice. J. natn. Cancer Inst., 46, 1.

WOODS, D. A. (1969) Influence of Antilymphocyte

Serum on DMBA Induction of Oral Carcinomas.
Nature, Lond., 224, 276.

				


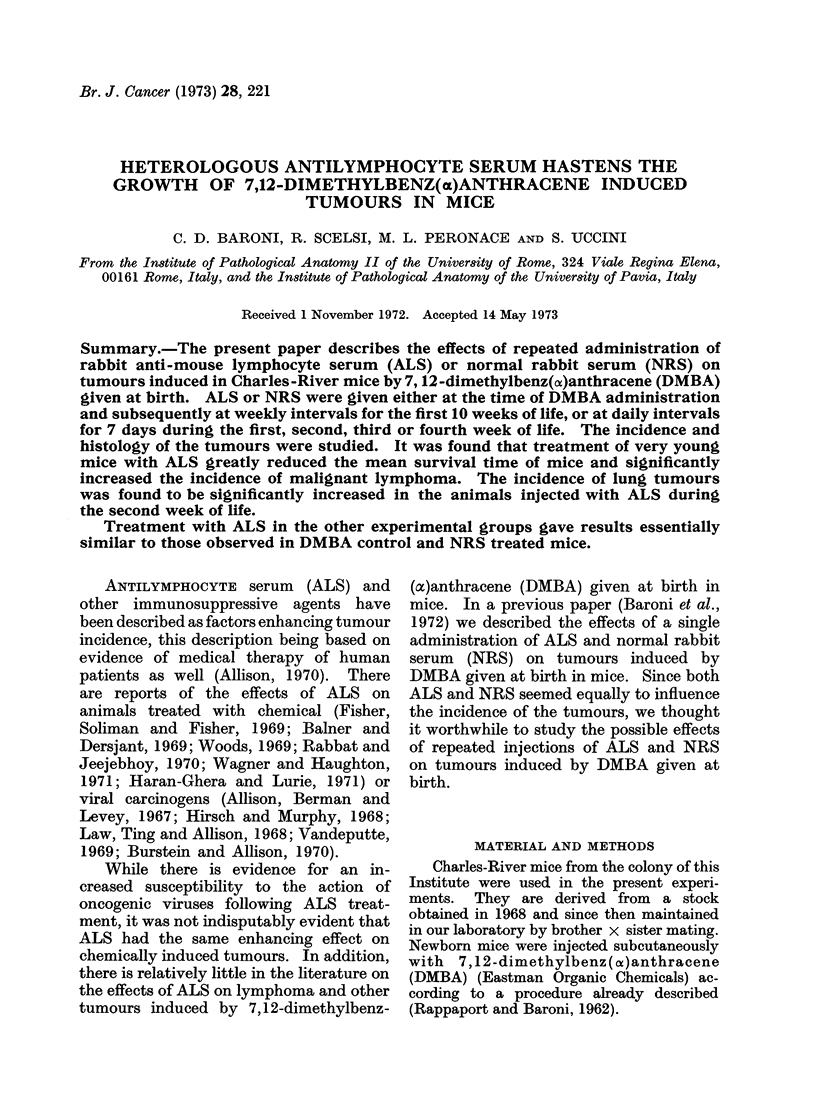

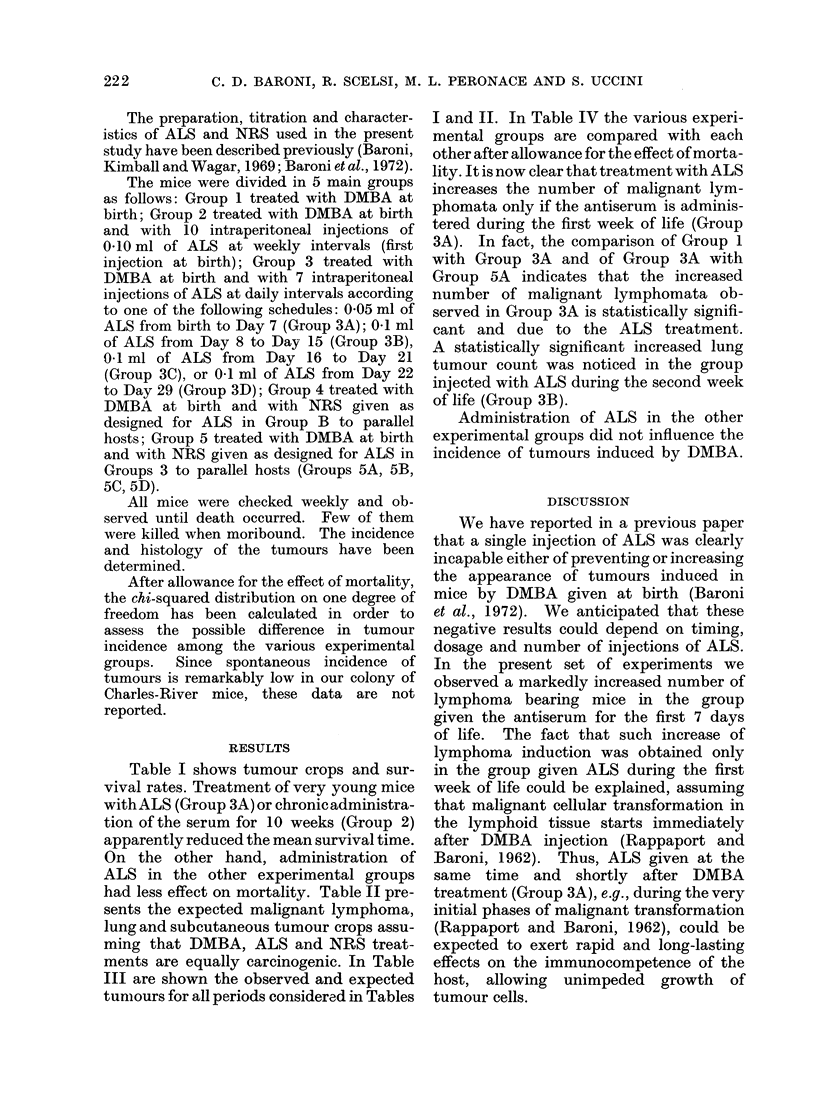

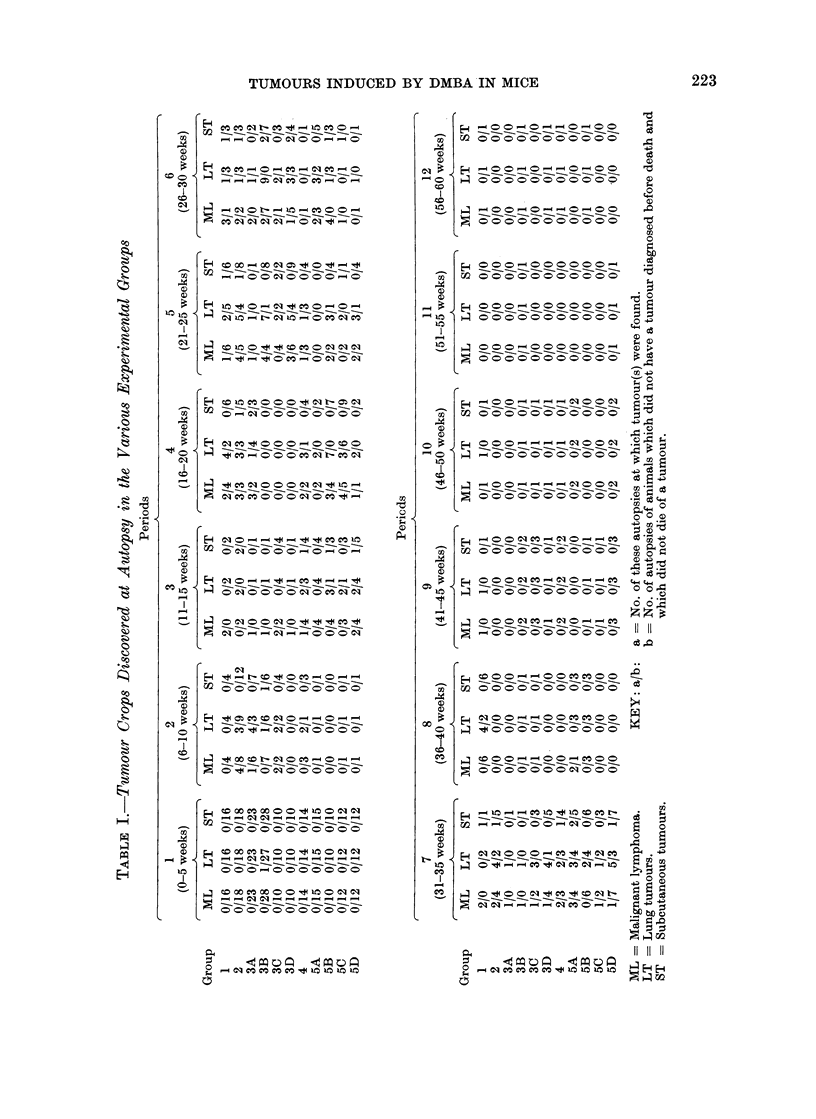

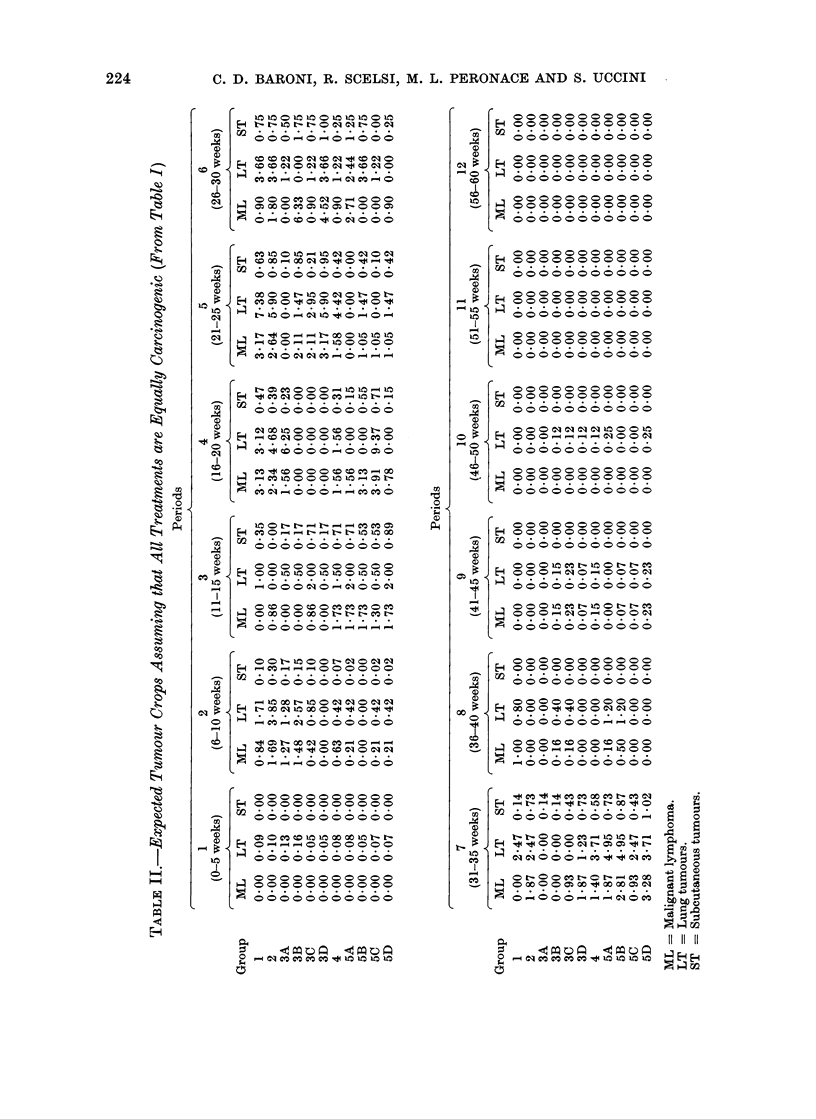

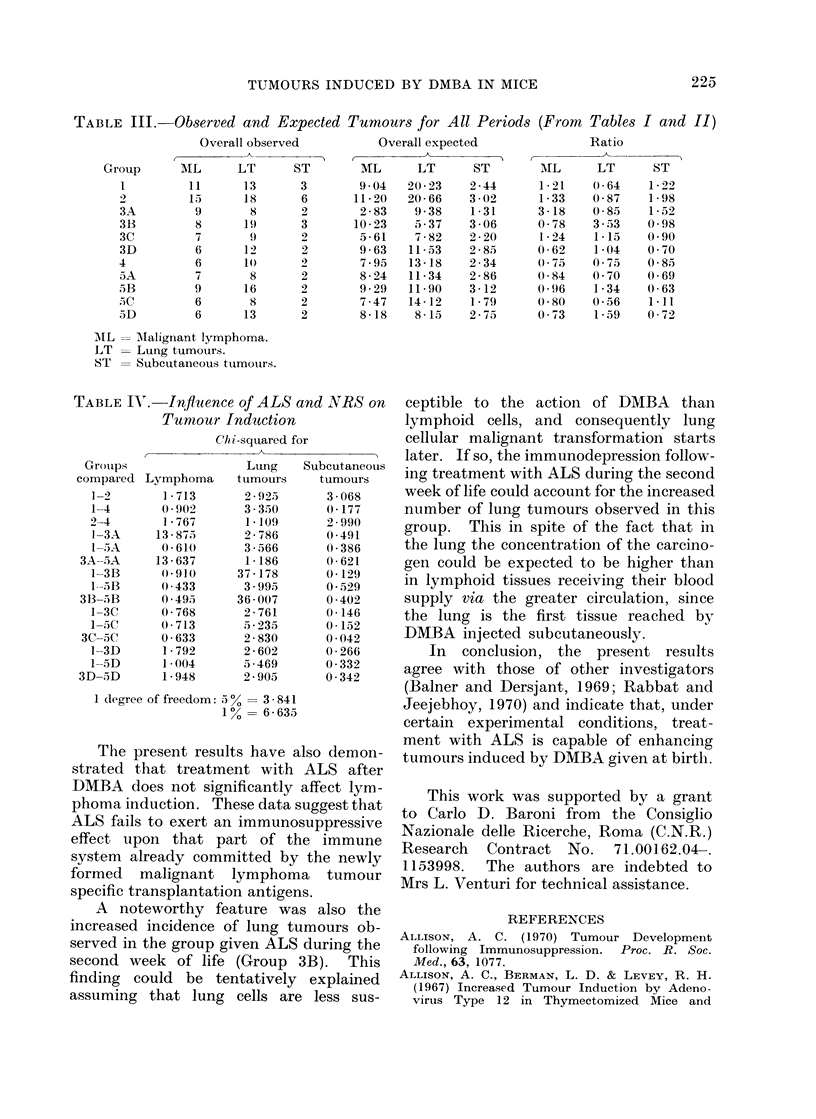

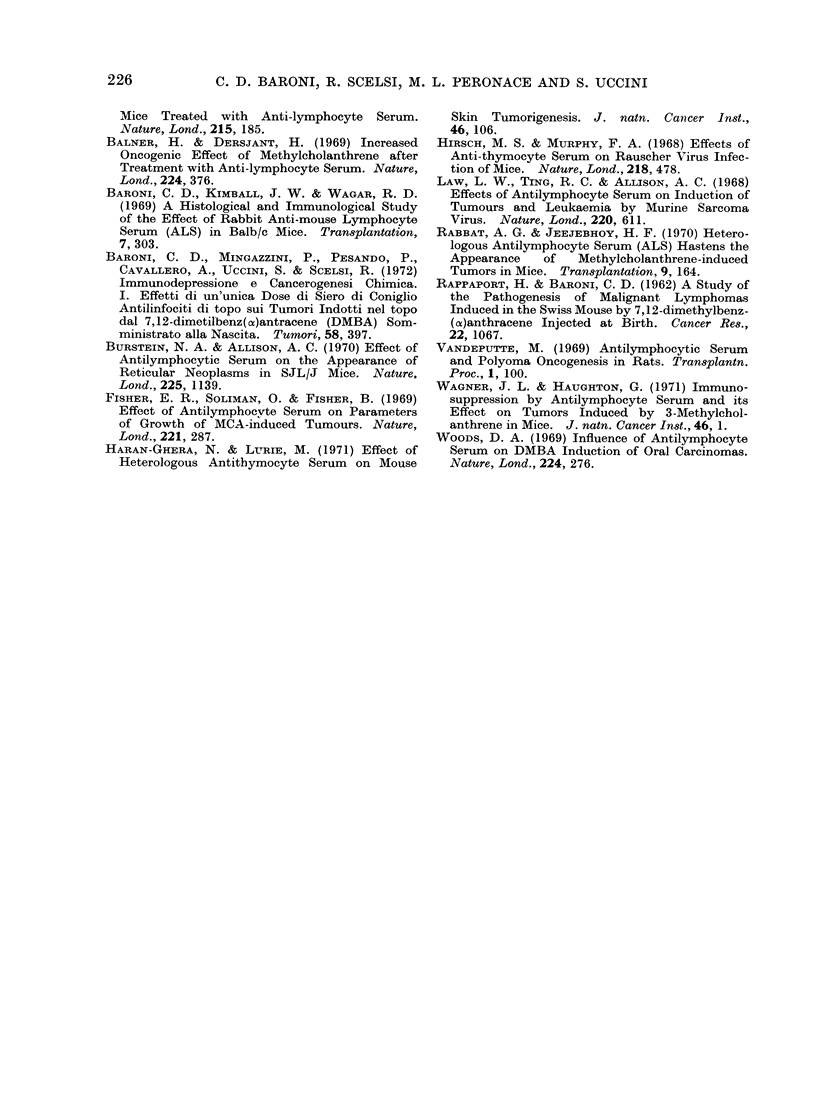


## References

[OCR_00708] Allison A. C., Berman L. D., Levey R. H. (1967). Increased tumour induction by adenovirus type 12 in thymectomized mice and mice treated with anti-lymphocyte serum.. Nature.

[OCR_00703] Allison A. C. (1970). Tumour development following immunosuppression.. Proc R Soc Med.

[OCR_00718] Balner H., Dersjant H. (1969). Increased oncogenic effect of methylcholanthrene after treatment with anti-lymphocyte serum.. Nature.

[OCR_00724] Baroni C. D., Kimball J. W., Ward E. N., Wagar R. D. (1969). A histological and immunological study of the effect of rabbit anti-mouse lymphocyte serum in BALB-c mice.. Transplantation.

[OCR_00731] Baroni C. D., Mingazzini P., Pesando P., Cavallero A., Uccini S., Scelsi R. (1972). Effetti di un'unica dose di siero antilinfocitico sui tumori indotti nel topo dal 7,12-dimentilbenz(a)antracene iniettato alla nascita.. Tumori.

[OCR_00740] Burstein N. A., Allison A. C. (1970). Effect of antilymphocytic serum on the appearance of reticular neoplasms in SJL-J mice.. Nature.

[OCR_00746] Fisher E. R., Soliman O., Fisher B. (1969). Effect of antilymphocyte serum on parameters of growth of MCA-induced tumours.. Nature.

[OCR_00759] Hirsch M. S., Murphy F. A. (1968). Effects of anti-thymocyte serum on Rauscher virus infection of mice.. Nature.

[OCR_00770] Rabbat A. G., Jeejeebhoy H. F. (1970). Heterologous antilymphocyte serum (ALS) hastens the appearance of methylcholanthrene-induced tumours in mice.. Transplantation.

[OCR_00764] Taylor R. B. (1968). Immune paralysis of thymus cells by bovine serum albumin.. Nature.

[OCR_00783] Vandeputte M. (1969). Antilymphocytic serum and polyoma oncogenesis in rats.. Transplant Proc.

[OCR_00788] Wagner J. L., Haughton G. (1971). Immunosuppression by antilymphocyte serum and its effect on tumors indduced by 3-methylcholanthrene in mice.. J Natl Cancer Inst.

[OCR_00794] Woods D. A. (1969). Influence of antilymphocyte serum on DMBA induction of oral carcinomas.. Nature.

